# Two Genetic Variations in the IRF8 region are associated with Behçet’s disease in Han Chinese

**DOI:** 10.1038/srep19651

**Published:** 2016-01-22

**Authors:** Yanni Jiang, Hong Wang, Hongsong Yu, Lin Li, Dengfeng Xu, Shengping Hou, Aize Kijlstra, Peizeng Yang

**Affiliations:** 1 The First Affiliated Hospital of Chongqing Medical University, Chongqing Key Laboratory of Ophthalmology and Chongqing Eye Institute, Chongqing, P R China; 2 Beijing Tongren Eye Center, Beijing Key Laboratory of Ophthalmology and Visual Science, Beijing Tongren Hospital, Capital Medical University, Beijing, P R China; 3 University Eye Clinic Maastricht, Maastricht, The Netherlands

## Abstract

Several modulatory factors in the TLR signaling pathway including IRF3, IRF7, IRF8, TRIM20, MYD88 and NF-κB1 have been associated with autoimmune disease. In this study, we investigated the association of 13 SNPs for these genes with Behçet’s disease (BD) and Vogt-Koyanagi-Harada (VKH) syndrome using a polymerase chain reaction restriction fragment length polymorphism (PCR-RFLP) assay. Haplotype and linkage disequilibrium (LD) analysis were performed by Haploview4.2. IRF8 mRNA expression and cytokine production was tested by real-time PCR and ELISA. Two SNPs near IRF8 were associated with BD (for rs17445836 GG genotype, Pc = 9.56 × 10^−8^, OR = 2.044; for rs11642873 AA genotype, Pc = 9.24 × 10^−7^, OR = 1.776). No significant association was found for the 13 SNPs tested with VKH syndrome. Haplotype analysis of the two positive SNPs revealed that the AG haplotype was significantly increased in BD patients (Pc = 2.60 × 10^−8^, OR = 1.646). Functional studies revealed an increased mRNA expression of IRF8 and IFN-γ production and a decreased production of IL-10 in rs17445836 carriers with the GG genotype. Increased expression of IRF8 as well as IFN-γ production and a decreased production of IL-10 were found in individuals carrying the rs11642873/AA genotype. In conclusion, this study indicates that IRF8 may contribute to the genetic susceptibility of BD by regulating IRF8 expression and cytokine production.

Uveitis is a sight threatening disease that can be caused by infectious or non-infectious mechanisms^1^. The non-infectious uveitis entities often have an autoinflammatory or autoimmune pathogenesis and are difficult to treat^2^. Understanding of the immunopathogenic mechanisms involved in uveitis may lead to novel therapeutic approaches. Analysis of the association of gene mutations encoding for factors of the immune system has markedly increased our understanding in the inflammatory pathways involved in uveitis^3^. Most of these studies have involved the study of common well defined uveitis entities such as acute anterior uveitis, Behçet’s disease (BD) and Vogt-Koyanagi–Harada syndrome (VKH)^4^.

Evidence is now becoming available implicating innate immune system triggers as a cause of the autoimmune or autoinflammatory mediated uveitis entities mentioned above^5^. The inflammatory response caused by these innate triggers, which may include microbial pathogens, is mediated via an interaction with Toll-like receptors (TLRs). TLRs have now been identified to have a critical role in the pathogenesis of various inflammatory diseases including uveitis^6^. The expression and function of various TLRs have been shown to be altered in BD^7^,^8^,^9^. Successful treatment of BD patients with anti TNF antibodies was furthermore associated with a decreased gene expression of TLR2^10^. These studies have recently been extended by showing that certain TLR gene polymorphisms and their gene copy numbers are significantly associated with the risk to develop a uveitis entity such as BD^11^,^12^. However, some studies were not able to show an association between BD and TLR gene polymorphisms^13^,^14^. These discrepancies might be due to the fact that earlier studies were underpowered or could be due to ethnic differences.

A role for TLR signaling elements in the pathogenesis of autoimmune disease has recently emerged^15^, although the role of this pathway in uveitis is not yet clear. The tripartite motif (TRIM) protein family, has been shown to regulate downstream signaling elements in the Toll-like receptor (TLR) pathway, subsequently leading to inflammatory cytokine synthesis^16^.

Various members of the TRIMs family, such as TRIM20, which is also called Mediterranean fever (MEFV) gene, as well as TRIM21 are associated with autoimmune diseases^17^,^18^. In the TRIM-mediated TLR pathway, TRIM21 can regulate the stabilization of diverse interferon regulatory factor (IRF) family members, such as IRF3/7/8, through a polyubiquitynation effect to accelerate the degradation of IRFs, thereby restricting the production of type I IFN. Furthermore, TRIM20 interacts with the Nuclear Factor-κB (NF-κB) P65/P50 subunit, thus promoting NF-κB production^19^. Myeloid differentiation factor 88 (MYD88) is another protein that participates in the TRIM-mediated TLR pathway^19^. Lately, gene polymorphisms of the participants in the TRIM-mediated TLR pathway, such as TRIM20, TRIM21, IRF3, IRF7, IRF8, NF-κB1-encoding P50 protein and MYD88 have been identified as risk factors that can predispose to a variety of autoimmune and autoinflammatory disorders^20^,^21^,^22^,^23^,^24^,^25^,^26^. Their possible role in uveitis has not yet been studied and was therefore the subject of the study presented here. We tested the association of six genes that are involved in the TRIM-mediated TLR pathway in BD and VKH patients with uveitis. Of the tested elements, only IRF8 was shown to be associated with susceptibility to uveitis in BD patients, whereby functional studies showed that the risk gene was able to regulate IRF8 expression as well as IFN-γ and IL-10 production.

## Results

### The clinical characteristics of BD and VKH patients

The detailed clinical characteristics of the enrolled BD and VKH patients are shown in Supplementary Table 2. In the normal controls, all the genotype frequencies of the 13 chosen SNPs did not deviate from the Hardy–Weinberg equilibrium.

### Genotype and allele frequencies of involved SNPs in the first phase

In the first stage study, 13 SNPs were genotyped in 384 BD and 384 VKH patients as well as 576 healthy controls. Significant differences among BD patients and healthy controls were detected in rs17445836 and rs11642873 of IRF8. The frequency of the AG genotype of rs17445836 in BD patients was decreased significantly (P = 0.001, Pc = 0.039, OR = 0.498) (Table 1). The frequencies of the AA genotype and A allele for rs11642873 showed a significant increase in BD patients (P = 8.72 × 10^−6^, Pc = 3.40 × 10^−4^, OR = 2.224; P = 8.09 × 10^−6^, Pc = 1.05 × 10^−4^, OR = 2.141, respectively). However, the AC genotype was significantly decreased in BD patients (P = 3.25 × 10^−5^, Pc = 1.27 × 10^−3^, OR = 0.472) in rs11642873. No significant differences in the other 11 SNPs were detected between BD patients and healthy individuals (Supplementary Table 3). None of the 13 SNPs showed a statistically significant association with VKH (Supplementary Table 4).

### Genotype and allele frequencies of involved SNPs in the replication phase and combined study

To confirm the associations we found in the first stage, we enrolled 697 BD patients and 1490 healthy individuals for a replication stage test. We only tested the SNPs that gave a significant result in the first stage study. Increased frequencies were detected in the GG genotype and G allele in rs17445836 in BD patients compared to healthy controls (P = 4.74 × 10^−7^, Pc = 1.85 × 10^−5^, OR = 2.081; P = 6.56 × 10^−7^, Pc = 8.53 × 10^−6^, OR = 1.997, respectively), and decreased frequencies in the AG genotype and A allele (P = 1.08 × 10^−6^, Pc = 4.21 × 10^−5^, OR = 0.487; P = 6.56 × 10^−7^, Pc = 8.53 × 10^−6^, OR = 0.501, respectively). For rs11642873, the AA genotype and A allele showed significantly increased frequencies in BD patients (P = 4.30 × 10^−5^, Pc = 1.68 × 10^−3^, OR = 1.668; P = 1.04 × 10^−4^, Pc = 1.35 × 10^−3^, OR = 1.571, respectively) and decreased frequencies in the AC genotype and C allele (P = 4.85 × 10^−5^, Pc = 1.89 × 10^−3^, OR = 0.594; P = 1.04 × 10^−4^, Pc = 1.35 × 10^−3^, OR = 0.636, respectively) (Table 1).

The combined study also showed that the two SNPs rs17445836 and rs11642873 were significantly associated with BD (for rs17445836 GG genotype, P = 2.45 × 10^−9^, Pc = 9.56 × 10^−8^, OR = 2.044; for AG genotype, P = 3.14 × 10^−9^, Pc = 1.22 × 10^−7^, OR = 0.486; for G allele, P = 8.14 × 10^−9^, Pc = 1.06 × 10^−7^, OR = 1.932; for rs11642873 AA genotype, P = 2.37 × 10^−8^, Pc = 9.24 × 10^−7^, OR = 1.776; for AC genotype, P = 8.01 × 10^−8^, Pc = 3.12 × 10^−6^, OR = 0.570; for A allele, P = 4.64 × 10^−8^, Pc = 6.03 × 10^−7^, OR = 1.694) (Table 1).

### Haplotype analysis of rs17445836 and rs11642873

For linkage disequilibrium (LD) and haplotype analysis between rs17445836 and rs11642873, we included 1071 BD patients and 2054 normal controls, who had genotyping data in both IRF8 risk SNPs found in our study. All these analyses were performed by using Haploview4.2 software. The result revealed that the two SNPs were in linkage disequilibrium (D′ = 0.56, r^2^ = 0.23) (Supplementary Fig. 1). Therefore, we carried out the haplotype analysis following Haploview4.2 software between the two SNPs (rs11642873-rs17445836) in BD patients and normal controls. The result showed that the AG haplotype had a significantly increased frequency in BD patients as compared to normal controls, on the contrary, the frequency of the CA haplotype in BD patients was decreased significantly compared with normal controls (P = 6.41 × 10^−9^, Pc = 2.60 × 10^−8^, OR = 1.646; P = 5.00 × 10^−8^, Pc = 2.00 × 10^−7^, OR = 0.439, respectively) (Table 2). There was no significant difference concerning CG and AA haplotype frequency between BD patients and normal controls (Table 2).

### The influence of rs17445836 and rs11642873 on the expression of IRF8

**Figure 1:**
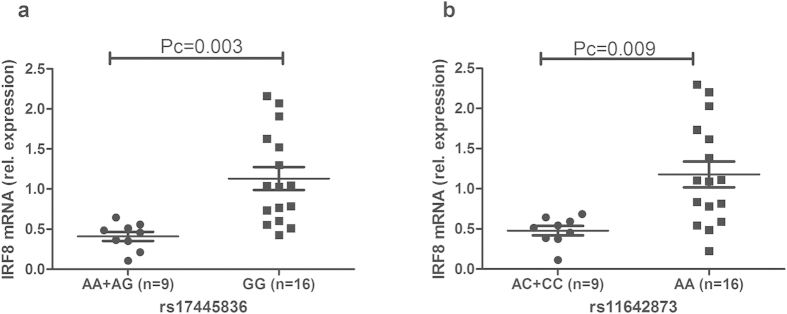
The influence of rs17445836 and rs11642873 on the relative expression of IRF8. The expression of IRF8 in PBMCs treated with anti-CD3/28 antibodies. PBMCs were obtained from healthy individuals with diverse genotypes of rs17445836 (**a**) and rs11642873 (**b**). Data show the mean ± SD. Pc: Bonferroni corrected p value, multiplied by 3.

As the results showed that two SNPs of IRF8 were associated with BD, we carried out the following experiments to explore whether IRF8 mRNA expression was affected by the different genotypes of rs17445836 and rs11642873. IRF8 mRNA expression was first analyzed by real-time PCR analysis in unstimulated PBMCs obtained from 25 unrelated genotyped healthy individuals. No effect was observed and also a stimulation of the PBMCs with LPS did not reveal differences in IRF8 mRNA expression among the various genotypes (Supplementary Fig. 2). Stimulation of the PBMCs with anti-CD3/CD28 antibodies did show a genotype effect. In rs17445836, individuals with the GG genotype had a significantly increased IRF8 mRNA expression as compared to carriers of the AA/AG genotype, Pc = 0.003 (Fig. 1). Carriers of the AA genotype in rs11642873 also had a higher mRNA expression of IRF8 than those with the AC/CC genotype, (Pc = 0.009, Fig. 1).

### The influence of rs17445836 and rs11642873 on cytokine production

**Figure 2:**
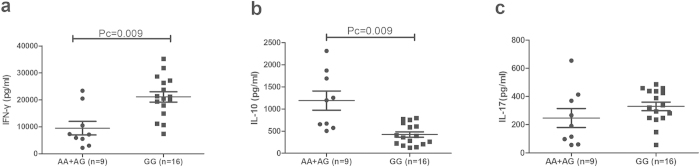
The influence of rs17445836 genotypes on cytokine production. The production of IFN-γ (**a**), IL-10 (**b**), IL-17 (**c**) in PBMCs obtained from healthy genotype controls. PBMCs were treated with anti-CD3/28 antibodies. Data show the mean ± SD. Pc: Bonferroni corrected p value, multiplied by 3.

**Figure 3:**
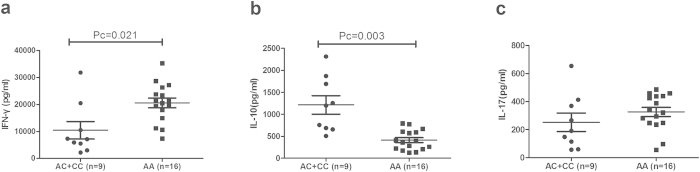
The influence of rs11642873 genotypes on cytokine production. The production of IFN-γ (**a**), IL-10 (**b**), IL-17 (**c**) in PBMCs treated with anti-CD3/28 antibodies. PBMCs were obtained from healthy genotyped controls. Data show the mean ± SD. Pc: Bonferroni corrected p value, multiplied by 3.

As the aforementioned results revealed that different genotypes of rs17445836 and rs11642873 affected the expression of IRF8, we performed a further study to explore whether the various genotypes of these two SNPs could affect cytokine production in PBMCs treated with anti-CD3/CD28. We examined three CD4 + T cell signature cytokines, IL-10, IL-17 and IFN-γ, which were also reported to participate in the development of BD^27^. The results revealed that the IFN-γ production by PBMCs in rs17445836 GG genotype carriers (21105 ± 7707 pg/ml) was higher than found in carriers with the AA/AG genotype (9506 ± 7494 pg/ml, Pc = 0.009). Inversely, GG genotype carriers (424 ± 241 pg/ml) showed a decreased production of IL-10 compared with AA/AG genotype carriers (1191 ± 649 pg/ml, Pc = 0.009). When examining the effect of rs11642873 genotypes on cytokine expression, we found an increased IFN-γ production in AA carriers (20580 ± 7199 pg/ml) compared with AC/CC carriers (10440 ± 9653 pg/ml, Pc = 0.021). On the contrary, the IL-10 production in CC carriers (411 ± 227 pg/ml) was decreased compared with AC/CC carriers (1213 ± 629 pg/ml; Pc = 0.003). No effect of genotype on IL-17 production was found when analyzing the various IRF8 rs17445836 and rs11642873 genotypes (Figs 2 and 3). Furthermore, some SNPs of IL-10 were associated with BD in Chinese Han population in previous studies^28^,^29^. So we tested two SNPs (rs1800871 and rs3021094) in the 25 unrelated genotyped healthy individuals used in the functional study to investigate the influence of SNPs on the production of IL-10. Unfortunately, the result revealed that there was no significant association between these two SNPs and different genotypes of IRF8/ rs17445836 and IRF8/ rs11642873 (Supplementary Tables 5 and 6).

## Discussion

We carried out a two-stage case control study to investigate the association of 13 SNPs of TRIM20, IRF3, IRF7, IRF8, MYD88 and NF-κB1 in two different uveitis entities. The results revealed that the GG genotype and G allele of IRF8/rs17445836, and the AA genotype and A allele for IRF8/rs11642873 confer a disease risk for BD. Haplotype analysis showed that AG haplotype carriers have a higher risk for BD, whereas CA haplotype carriers have a decreased risk for BD. Healthy subjects with the GG genotype in rs17445836 and AA genotype in rs11642873 displayed an increased IRF8 expression compared to the other genotypes. Both genotypes were also associated with an increased IFN-γ and a reduced IL-10 release. No significant association of the other 11 SNPs with BD was found and none of the 13 SNPs tested showed an association with VKH syndrome. The discordance between the IRF8 association with BD but not with VKH may be due to the different immunologic mechanisms leading to these two diseases. BD is regarded as an auto-inflammatory disease, while VKH is considered an autoimmune disease directed against melanocytes^30^.

IRF8, which is located in 16q24.1, belongs to the IFN regulatory factor (IRF) family, and takes part in TLR signaling, thus playing a role in the innate immune response^31^. Recent reports indicated that IRF8 can regulate the development of macrophages, DCs, B lymphocytes, as well as T lymphocytes^32^,^33^. Earlier reports revealed that IRF8 polymorphisms were associated with diverse autoimmune and autoinflammatory diseases^24^,^26^,^34^,^35^,^36^,^37^,^38^. Rs17445836 and rs11642873 are located in the downstream region of the IRF8 gene and are in linkage disequilibrium. How a downstream locus outside of the IRF8 coding region affects biological function is not clear but could be due to a linkage disequilibrium with other loci. Our finding showing a significant association between the GG genotype and G allele of rs17445836 with BD is in agreement with earlier American studies in systemic lupus erythematosus (SLE)^36^. For rs11642873, we found that the frequency of the AA genotype and A allele were significantly elevated in BD patients, and a similar result has been reported for Systemic Sclerosis in Caucasians^34^. We also tested four other SNPs of IRF8 (rs2280381, rs11644034, rs11648084, and rs925994), but could not detect a significant association with BD. These results suggest that the rs17445836 GG genotype and G allele and rs11642873 AA genotype and A allele are two predisposing factors for these autoimmune and autoinflammatory diseases. Haplotype analysis of rs11642873 and rs17445836, showed that the AG haplotype was the susceptibility haplotype for BD and that the CA haplotype was the protective one.

As the aforementioned results showed that rs17445836 and rs11642873 were significantly associated with BD, we performed a further study to examine whether the two SNPs could influence the expression of cytokines involved in the development of BD. As the patients were not a homogenous group due to differences in the degree of the inflammatory process and the treatment with immunosuppressive drugs, we only investigated the influence of rs17445836 and rs11642873 in healthy controls. We did not find significant changes of the IRF8 expression in PBMCs without stimulation or when the cells were stimulated with LPS among different genotypes of these two SNPs. However, an increased IRF8 mRNA expression was observed in rs17445836 GG genotype individuals in contrast to AG/AA genotype individuals, and in rs11642873 AA genotype cases compared to AC/CC genotype cases when PBMCs were treated with anti-CD3/CD28 antibodies to simulate antigen presentation. This confirms earlier studies showing that IRF8 is rapidly induced following antigen stimulation^39^ and it shows that IRF8 may be required for the regulation of T cell effector functions. Our findings also indicate that the IRF8 genotype may have a more profound effect on T cells than on macrophages and dendritic cells, since the stimulation with LPS showed no effect. Our findings that the IRF8 rs17445836 genotype can affect the IRF8 level is in accordance with recent findings showing an effect of rs17445836 on IRF8 expression in SLE patient B cells^36^. The studies mentioned above mainly dealt with circumstances leading to an altered intracellular IRF8 level. IRF8 function itself is triggered via an interaction of single stranded DNA or RNA with endosomal associated TLR3 and TLR7-9. We did not test these ligands and further studies are needed to investigate the response of various IRF8 genotypes following stimulation with these agonists.

We investigated three types of T cell cytokines, which have been implicated to play a role in the pathogenesis of BD, namely IFN-γ, IL-10 and IL-17^40^. A significant increase was observed in the production of IFN-γ in individuals carrying the rs17445836 GG genotype as compared to the AG/AA genotype, and in individuals carrying the rs11642873 AA genotype compared with individuals having the AC/CC genotype. In addition, we found a significant reduction of IL-10 in carriers of the rs17445836 GG genotype compared to individuals having the AG/AA genotype, as well as in cases with the rs11642873 AA genotype compared with individuals having the AC/CC genotype. Our results are in agreement with the dogma that IL-10 is an anti-inflammatory cytokine and that IFN-γ acts as a pro-inflammatory cytokine^27^,^41^. The elevated production of IFN-γ and diminished production of IL-10 in individuals with the rs17445836 GG genotype and rs11642873 AA genotype are in line with the predisposing role of the two genotypes for BD. These findings are in agreement with a recent study showing that IL-10 was upregulated in the retina of IRF8-deficient mice during experimental autoimmune uveitis, while IFN-γ and IL-17 were downregulated^42^. We did not find an effect on the production of IL-17 in the three genotypes of either rs17445836 or rs11642873. The discrepancy with the mice study mentioned above might be due to species differences.

The IRF8 genotype was shown to affect IL-10 expression. Since SNPs of IL-10 have been shown to be associated with BD^28^,^29^, we examined whether certain IL-10 genotypes showed a higher frequency within a certain IRF8 genotype group. A small scale experiment did not reveal an interaction between the IL-10 and IRF8 genotypes (Supplementary Tables 5 and 6).

IRF3 and IRF7 are two other members of IRF family, inducing type I IFN expression, taking part in innate immunity^43^. Previous studies identified that rs2280381/IRF3^22^, rs1131665/IRF7^44^ and rs1061501/IRF7^45^ were associated with SLE in American and Asian population. We excluded the polymorphism of rs1131665, due to the fact that no data were available in the Chinese Han NCBI dbSNP database. We therefore only investigated rs2280381/IRF3 and rs1061501/IRF7. No significant association for rs1061501 and rs2280381 and BD or VKH was found.

TRIM20, also called Mediterranean Fever (MEFV) gene, which is located on chromosome 16p13, is regarded as a responsible gene for another systemic autoinflammatory disorder: Familial Mediterranean Fever (FMF) disease^46^,^47^. It encodes protein Pyrin which is considered to be connected with NF-κB-related inflammation^46^,^47^. There are five common mutations of TRIM20 (E148Q, M680I, M694V, V726A, P369S) that have been widely studied in many autoinflammatory diseases, including BD^48^. We did not investigate the five mutations because the MAF of E148Q was lower than 0.05 and there was no data available in the Chinese Han NCBI dbSNP database for the other four mutations (M680I, M694V, V726A, P369S). SNPs of TRIM20 (rs224225 and rs224217) were shown to be associated with inflammatory bowel disease^49^, and rs224204 was associated with juvenile idiopathic arthritis in Caucasians^21^. However, none of these SNPs were found to be associated with BD or VKH syndrome in our study.

MYD88 protein is a vital signal transducing adaptor protein in the TLR pathway due to its interaction with the interleukin-1 receptor-associated kinase^50^. One SNP of MYD88, namely rs7744 was reported to be associated with ulcerative colitis^25^. Nevertheless, no significant associations of rs7744 with BD and VKH were revealed in our study.

NF-κB1 encodes protein P50, belonging to Nuclear factor kappa B (NF-κB) family. P50 binds with P65, another member of NF-κB, and the two elements compose a biologically active heterodimer, which can interact with the promoter sequences of IL-1 and IFN-γ thereby regulating their transcription^51^. SNPs of NF-κB1 were reported to be associated with BD in Turkey (rs28362491)^52^ and with ulcerative colitis (rs3774959)^26^. Rs28362491 was excluded in our study because no data was available in the Chinese Han NCBI dbSNP database. In our study, rs3774959 revealed no significant association with BD or VKH disease.

The differences between our results and the other studies concerning associations with the factors mentioned above may be due to differences in the pathogenesis of these diseases, different ethnicity or due to sample sizes.

We assured the validity of polymorphism analysis results by the following steps. Firstly, all the candidate SNPs were selected based on three standards as mentioned earlier. Secondly, the diagnoses of BD and VKH syndrome were strictly based on the guidelines of the International Study Group and the First International Workshop^53^,^54^. Finally, we randomly selected 5% of all samples to perform direct sequencing to validate or genotyping method. All our genotyping results were in agreement with the direct sequence data.

Our study has some limitations. First, our participants were all Chinese Han and confirmation of our results by multicenter trials but also in other ethnic groups is necessary. All the BD and VKH syndrome patients came from an ophthalmology clinic. Since BD and VKH are multisystem diseases, further studies should also involve patients coming from other clinical departments. Furthermore, we only tested a limited number of SNPs of IRF3, IRF7, IRF8, TRIM20, MYD88 and NF-κB1 in BD and VKH disease, and we cannot exclude that other yet not tested SNPs may show an association with these two diseases. One should also note that the risk association found is small since 83% of healthy Chinese are rs17445836 GG carriers as compared to 91% of the BD patients. Similar differences are found for rs11642873 CC (78% in controls versus 86% in BD). We would finally like to note that the reported association was observed in BD uveitis and should not be extrapolated to other uveitis entities.

In conclusion, our results revealed that IRF8 (rs17445836 and rs11642873) polymorphisms and haplotypes were associated with BD. In addition, our results suggested that the genetic predisposition found for BD may due to mechanisms that are involved in the regulation of IRF8 mRNA expression and cytokine production.

## Materials and Methods

### Study subjects

A two-stage case-control study was carried out. In the first stage, 384 BD patients, 384 VKH patients and 576 normal subjects were enrolled. These patients had visited the Zhongshan Ophthalmic Center of Sun Yat-sen University (Guangzhou, China) and the First Affiliated Hospital of Chongqing Medical University (Chongqing, China) from June 2006 to October 2014. The second stage included another independent set of 697 BD patients and 1490 healthy individuals. All of the subjects were of Chinese Han descent. The diagnosis of BD was based on the criteria of the International Study Group^53^. All the enrolled BD patients had uveitis, in which 1028 patients had panueitis, whereas only 53 BD patients had anterior uveitis. The diagnosis for VKH was according to the definition that was made during the First International Workshop on VKH^54^.

### Ethical considerations

All the participants signed an informed consent form before enrolling into this study. The investigation protocols were approved by the Clinical Research Ethics Committee of the Zhongshan Ophthalmic Center of Sun Yat-sen University and the First Affiliated Hospital of Chongqing Medical University. All experiments were performed according to the approved guidelines and regulations. The study was performed according to the tenets of the Declaration of Helsinki.

### SNPs selecting

We selected SNPs based on three standards: 1. Available literature reports on the possible association of the relevant SNP with an autoimmune or auto-inflammatory disease. 2. Presence of allele or genotype data in the Chinese Han National Center of Biotechnology Information (NCBI) database of single nucleotide polymorphisms (dbSNP). 3. The minor allele frequencies (MAF) in our population had to be greater than 0.05, since a MAF ≤0.05, would lack statistical power with the used patient sample size. Following these standards, we excluded rs660 in TRIM21, rs1131665 in IRF7, three SNPs (rs224222, rs224224, rs224223 and five common mutations^48^) in TRIM20 and rs28362491 in NF-κB1, because no data were available in the Chinese Han NCBI dbSNP database. Therefore we selected 13 SNPs as candidate SNPs, namely one SNP (rs2304204) for IRF3, one SNP (rs1061501) for IRF7, six SNPs (rs11644034, rs925994, rs11648084, rs2280381, rs11642873 and rs17445836) for IRF8, one (rs7744) for MYD88, one (rs3774959) for NF-κB1 and three SNPs (rs224204, rs224217, and rs224225) for TRIM20.

### Genotyping

DNA samples were extracted from the peripheral blood in tubes containing Ethylene Diamine Tetraacetic Acid (EDTA) anticoagulant, and isolated with the QIAamp DNA Blood Mini Kit (Qiagen, Valencia, CA). DNA sequences were amplified by PCR using the primers shown in Table S1. The restriction enzymes used to digest the PCR products are listed in Supplementary Table 1. The digestion products were separated in 4% agarose gel for four SNPs (rs2304204, rs7744, rs11644034, rs17445836) and 5% agarose gel for the other SNPs. The successful genotyping rate for the different SNPs ranged from 97.9% to 100%. To validate the genotyping method employed in our study, we randomly selected 5% of the samples for direct sequencing that was carried out by Sangon Biotech Company (Shanghai, China). The allele and genotype frequency of SNPs in IL-10 that have been shown to affect IL-10 expression (rs1800871, and rs3021094), was analyzed by direct sequencing performed in the Sangon Biotech Company (Shanghai, China).

### Cell isolation and culture

Peripheral blood mononuclear cells (PBMCs) were separated from venous blood samples obtained from 25 healthy genotyped controls by Ficoll-Hypaque density-gradient centrifugation. The separated PBMCs were placed in 96 well-plates (2 × 10^6^ cells/well), and cultured in RPMI 1640 medium. PBMCs were treated with anti-CD3 antibody (Miltenyi Biotec, Palo Alto, CA) and anti-CD28 (Miltenyi Biotec, Palo Alto, CA) antibody (5:1) to simulate antigen presentation for 3 days, or were cultured in LPS (Miltenyi Biotec, Palo Alto, CA) to simulate an inflammatory signal (100ng/mL, Sigma, Missouri, USA) for 1 day.

### Real-time PCR

Total RNA was extracted from PBMCs using TRIzol (Invitrogen, Carlsbad, CA). The real-time PCR was accomplished on a 7500 system (ABI, Foster City, CA, USA). We selected β-actin as the internal reference gene. The IRF8 expression was tested by using the following primers: forward 5′-CCAGCCAGTTCTTCCGA-3′ and reverse 5′-CCTCTTCTGCCAGTTG CC-3′. The primers for β-actin were as follows: forward 5′-GGATGCAGAAGGAGATCACTG -3′ as well as reverse 5′-CGATCCACA CGGAGTACTT-3′. The relative expression level was computed using 2 ^−ΔΔCt^ method.

### ELISA

The supernatants of stimulated PBMCs were collected to test the concentration of cytokines (IFN-γ, IL-10, and IL-17) by the human Duoset ELISA development kit (R&D Systems, Minneapolis, USA).

### Statistical analysis

The chi-square or Fisher exact test (for N < 40) were applied to compare genotype and allele frequencies and Hardy-Weinberg equilibrium detection. Odds ratios (OR) and 95% confidence intervals (95% CI) were calculated to evaluate the disease risk. IRF8 expression and cytokine data between different genotype groups were evaluated using the Mann–Whitney U test. In case the data were normally distributed, we used the independent samples t-test. All statistical tests were performed using SPSS 17.0 (SPSS Inc., Chicago, USA). Linkage disequilibrium (LD) and haplotype analysis between the two positive SNPs (rs11642873 and rs17445836) of the IRF8 gene were performed by the Haploview4.2 software. R^2^ and the absolute value of Lewontin’s /D’/ was used to estimate LD between the two SNPs^55^. Statistical analysis of the haplotype data was calculated by chi-square test. To correct the p value for multiple comparisons, a Bonferroni correction was applied. P values obtained for genotype, allele and haplotype analysis were multiplied by a factor 39 for the genotypes, by a factor 13 for allele and by a factor 4 for haplotype comparisons. P values obtained for IRF8 mRNA and cytokine comparisons were multiplied by 3. P values obtained for genotype and allele analysis for SNPs in IL-10 were multiplied by a factor 6 for the genotypes and by a factor 2 for the alleles. A P-corrected (Pc) value <0.05 was regarded as significant.

## Additional Information

**How to cite this article**: Jiang, Y. *et al.* Two Genetic Variations in the IRF8 region are associated with Behçet's disease in Han Chinese. *Sci. Rep.* **6**, 19651; doi: 10.1038/srep19651 (2016).

## Supplementary Material

Supplementary Information

## Figures and Tables

**Table 1:** Influences of IRF8/rs17445836 and IRF8/rs11642873 on BD risk.

Stage	Genotype Allele	Case N (freq.)	Control N (freq.)	P value	Pc value	OR (95% CI)
rs17445836						
Stage I	A A	2(0.005)	1(0.002)	0.353	NS	2.963(0.268–32.794)
	A G	31(0.081)	85(0.151)	0.001	0.039	0.498(0.323–0.768)
	G G	349(0.914)	478(0.848)	0.003	NS	1.903(1.245–2.909)
	G	729(0.954)	1041(0.923)	0.007	NS	1.741(1.163–2.606)
	A	35(0.046)	87(0.077)	0.007	NS	0.574(0.384–0.860)
Stage II	A A	2(0.003)	10(0.007)	0.257	NS	0.426(0.093–1.949)
	A G	62(0.089)	249(0.167)	1.08 × 10^−6^	4.21 × 10^−5^	0.487(0.363–0.653)
	G G	633(0.908)	1231(0.826)	4.74 × 10^−7^	1.85 × 10^−5^	2.081(1.557–2.781)
	G	1328(0.953)	2711(0.910)	6.56 × 10^−7^	8.53 × 10^−6^	1.997(1.513–2.634)
	A	66(0.047)	269(0.090)	6.56 × 10^−7^	8.53 × 10^−6^	0.501(0.380–0.661)
Combined	A A	4(0.004)	11(0.005)	0.525	NS	0.691(0.220–2.175)
	A G	93(0.086)	334(0.163)	3.14 × 10^−9^	1.22 × 10^−7^	0.486(0.381–0.619)
	G G	982(0.910)	1709(0.832)	2.45 × 10^−9^	9.56 × 10^−8^	2.044(1.610–2.594)
	G	2057(0.953)	3752(0.913)	8.14 × 10^−9^	1.06 × 10^−7^	1.932(1.539–2.426)
	A	101(0.047)	356(0.087)	8.14 × 10^−9^	1.06 × 10^−7^	0.517(0.412–0.650)
rs11642873						
Stage I	A A	335(0.875)	433(0.758)	8.72 × 10^−6^	3.40 × 10^−4^	2.224(1.555–3.182)
	A C	48(0.125)	133(0.233)	3.25 × 10^−5^	1.27 × 10^−3^	0.472(0.329–0.676)
	C C	0	5(0.009)	0.066	NS	1.009(1.001–1.017)
	A	718(0.937)	999(0.875)	8.09 × 10^−6^	1.05 × 10^−4^	2.141(1.532–3.010)
	C	48(0.063)	143(0.125)	8.09 × 10^−6^	1.05 × 10^−4^	0.467(0.332–0.657)
Stage II	A A	590(0.858)	1166(0.783)	4.30 × 10^−5^	1.68 × 10^−3^	1.668(1.303–2.135)
	A C	92(0.134)	307(0.206)	4.85 × 10^−5^	1.89 × 10^−3^	0.594(0.462–0.765)
	C C	6(0.009)	16(0.011)	0.661	NS	0.810(0.316–2.079)
	A	1272(0.924)	2639(0.886)	1.04 × 10^−4^	1.35 × 10^−3^	1.571(1.249–1.977)
	C	104(0.076)	339(0.114)	1.04 × 10^−4^	1.35 × 10^−3^	0.636(0.506–0.801)
Combined	A A	925(0.864)	1609(0.781)	2.37 × 10^−8^	9.24 × 10^−7^	1.776(1.449–2.176)
	A C	140(0.131)	430(0.209)	8.01 × 10^−8^	3.12 × 10^−6^	0.570(0.463–0.701)
	C C	6(0.006)	21(0.010)	0.187	NS	0.547(0.220–1.359)
	A	1990(0.929)	3648(0.885)	4.64 × 10^−8^	6.03 × 10^−7^	1.694(1.400–2.050)
	C	152(0.071)	472(0.115)	4.64 × 10^−8^	6.03 × 10^−7^	0.590(0.488–0.714)

Pc: Bonferroni corrected p value, multiplied by 39/genotype and 13/allele. NS, not significant.

**Table 2:** The haplotype analysis of rs11642873 and rs17445836 in BD patients and controls.

Haplotype	Case (freq., %)	Control (freq., %)	P value	Pc value	OR (95% CI)
IRF8(rs11642873-rs17445836)					
A G	1942(90.7)	3512.6(85.5)	6.41 × 10^−9^	2.60 × 10^−8^	1.646(1.389–1.950)
C G	99(4.6)	239.4(5.8)	0.046	NS	0.783(0.616–0.996)
C A	53(2.5)	224.6(5.5)	5.00 × 10^−8^	2.00 × 10^−7^	0.439(0.324–0.595)
A A	48(2.2)	131.4(3.2)	0.031	NS	0.694(0.496–0.970)

Pc: Bonferroni corrected p value, multiplied by 4. NS, not significant.

## References

[b1] Lee R. W. *et al.* Autoimmune and autoinflammatory mechanisms in uveitis. Semin. Immunopathol. 36, 581–594 (2014). 24858699 10.1007/s00281-014-0433-9 PMC4186974

[b2] Larson T., Nussenblatt R. B. & Sen H. N. Emerging drugs for uveitis. Expert Opin. Emerg. Drugs 16, 309–322 (2011). 21210752 10.1517/14728214.2011.537824 PMC3102121

[b3] Rosenbaum J. T. & Kim H. W. Innate immune signals in autoimmune and autoinflammatory uveitis. Int. Rev. Immunol. 32, 68–75 (2013). 23360159 10.3109/08830185.2012.750132

[b4] Du L., Kijlstra A. & Yang P. Immune response genes in uveitis. Ocul. Immunol. Inflamm. 17, 249–256 (2009). 19657978 10.1080/09273940902999356

[b5] Willermain F. *et al.* Interplay between innate and adaptive immunity in the development of non-infectious uveitis. Prog. Retin. Eye Res. 31, 182–194 (2012). 22120610 10.1016/j.preteyeres.2011.11.004 PMC3288447

[b6] Chang J. H., McCluskey P. J. & Wakefield D. Recent advances in Toll-like receptors and anterior uveitis. Clin. Experiment. Ophthalmol. 40, 821–828 (2012). 22429223 10.1111/j.1442-9071.2012.02797.x

[b7] Seoudi N. *et al.* The role of TLR2 and 4 in Behcet’s disease pathogenesis. Innate Immun. 20, 412–422 (2014). 23940075 10.1177/1753425913498042

[b8] Liu X., Wang C., Ye Z., Kijlstra A. & Yang P. Higher expression of Toll-like receptors 2, 3, 4, and 8 in ocular Behcet’s disease. Invest. Ophthalmol. Vis. Sci. 54, 6012–6017 (2013). 23908180 10.1167/iovs.13-12159

[b9] Liang L. *et al.* IL-1beta triggered by peptidoglycan and lipopolysaccharide through TLR2/4 and ROS-NLRP3 inflammasome-dependent pathways is involved in ocular Behcet’s disease. Invest. Ophthalmol. Vis. Sci. 54, 402–414 (2013). 23211828 10.1167/iovs.12-11047

[b10] Keino H., Watanabe T., Taki W. & Okada A. A. Effect of infliximab on gene expression profiling in Behcet’s disease. Invest. Ophthalmol. Vis. Sci. 52, 7681–7686 (2011). 21862654 10.1167/iovs.11-7999

[b11] Fang J. *et al.* Association of TLR2 gene polymorphisms with ocular Behcet’s disease in a Chinese Han population. Invest. Ophthalmol. Vis. Sci. 54, 8384–8392 (2013). 24255044 10.1167/iovs.13-12878

[b12] Fang J. *et al.* Association Between Copy Number Variations of TLR7 and Ocular Behcet’s Disease in a Chinese Han Population. Invest. Ophthalmol. Vis. Sci. 56, 1517–1523 (2015). 25650422 10.1167/iovs.14-15030

[b13] Ben Dhifallah I., Lachheb J., Houman H. & Hamzaoui K. Toll-like-receptor gene polymorphisms in a Tunisian population with Behcet’s disease. Clin. Exp. Rheumatol. 27, S58–62 (2009). 19796535

[b14] Song G. G., Choi S. J., Ji J. D. & Lee Y. H. Toll-like receptor polymorphisms and vasculitis susceptibility: meta-analysis and systematic review. Mol. Biol. Rep. 40, 1315–1323 (2013). 23065292 10.1007/s11033-012-2175-x

[b15] Mills K. H. TLR-dependent T cell activation in autoimmunity. Nat. Rev. Immunol. 11, 807–822 (2011). 22094985 10.1038/nri3095

[b16] McNab F. W., Rajsbaum R., Stoye J. P. & O’Garra A. Tripartite-motif proteins and innate immune regulation. Curr. Opin. Immunol. 23, 46–56 (2011). 21131187 10.1016/j.coi.2010.10.021

[b17] Oke V. & Wahren-Herlenius M. The immunobiology of Ro52 (TRIM21) in autoimmunity: a critical review. J. Autoimmun. 39, 77–82 (2012). 22402340 10.1016/j.jaut.2012.01.014

[b18] Ozen S., Hoffman H. M., Frenkel J. & Kastner D. Familial Mediterranean fever (FMF) and beyond: a new horizon. Fourth International Congress on the Systemic Autoinflammatory Diseases held in Bethesda, USA, 6-10 November 2005. Ann. Rheum. Dis. 65, 961–964 (2006). 16606647 10.1136/ard.2006.052688 PMC1798229

[b19] Jefferies C., Wynne C. & Higgs R. Antiviral TRIMs: friend or foe in autoimmune and autoinflammatory disease? Nat. Rev. Immunol. 11, 617–625 (2011). 21866173 10.1038/nri3043

[b20] Tatari-Calderone Z., Luban N. L. & Vukmanovic S. Genetics of transfusion recipient alloimmunization: can clues from susceptibility to autoimmunity pave the way? Transfus. Med. Hemother. 41, 436–445 (2014). 25670931 10.1159/000369145 PMC4280455

[b21] Day T. G. *et al.* Autoinflammatory genes and susceptibility to psoriatic juvenile idiopathic arthritis. Arthritis Rheum. 58, 2142–2146 (2008). 18576390 10.1002/art.23604 PMC2688675

[b22] Akahoshi M. *et al.* Promoter polymorphisms in the IRF3 gene confer protection against systemic lupus erythematosus. LUPUS 17, 568–574 (2008). 18539711 10.1177/0961203308089340

[b23] Carmona F. D. *et al.* Novel identification of the IRF7 region as an anticentromere autoantibody propensity locus in systemic sclerosis. Ann. Rheum. Dis. 71, 114–119 (2012). 21926187 10.1136/annrheumdis-2011-200275 PMC3369428

[b24] Cunninghame Graham D. S. *et al.* Association of NCF2, IKZF1, IRF8, IFIH1, and TYK2 with systemic lupus erythematosus. PLoS Genet. 7, e1002341 (2011). 22046141 10.1371/journal.pgen.1002341 PMC3203198

[b25] Matsunaga K. *et al.* The *1244 A > G polymorphism of MyD88 (rs7744) is closely associated with susceptibility to ulcerative colitis. M*ol Med Rep* 9, 28–32 (2014). 24189845 10.3892/mmr.2013.1769

[b26] De Jager P. L. *et al.* Meta-analysis of genome scans and replication identify CD6, IRF8 and TNFRSF1A as new multiple sclerosis susceptibility loci. Nat. Genet. 41, 776–782 (2009). 19525953 10.1038/ng.401 PMC2757648

[b27] Raphael I., Nalawade S., Eagar T. N. & Forsthuber T. G. T cell subsets and their signature cytokines in autoimmune and inflammatory diseases. Cytokine (2014). 10.1016/j.cyto.2014.09.011 PMC4416069 25458968

[b28] Wu Z. *et al.* IL10 polymorphisms associated with Behcet’s disease in Chinese Han. Hum. Immunol. 75, 271–276 (2014). 24269690 10.1016/j.humimm.2013.11.009

[b29] Hu J. *et al.* Interleukin-10 gene polymorphisms are associated with Behcet’s disease but not with Vogt-Koyanagi-Harada syndrome in the Chinese Han population. Mol. Vis. 21, 589–603 (2015). 26015771 PMC4443582

[b30] McGonagle D. & McDermott M. F. A proposed classification of the immunological diseases. PLoS Med. 3, e297 (2006). 16942393 10.1371/journal.pmed.0030297 PMC1564298

[b31] Zhao J. *et al.* IRF-8/interferon (IFN) consensus sequence-binding protein is involved in Toll-like receptor (TLR) signaling and contributes to the cross-talk between TLR and IFN-gamma signaling pathways. J. Biol. Chem. 281, 10073–10080 (2006). 16484229 10.1074/jbc.M507788200

[b32] Tamura T. *et al.* IFN regulatory factor-4 and -8 govern dendritic cell subset development and their functional diversity. J. Immunol. 174, 2573–2581 (2005). 15728463 10.4049/jimmunol.174.5.2573

[b33] Yoshida Y. *et al.* The transcription factor IRF8 activates integrin-mediated TGF-beta signaling and promotes neuroinflammation. Immunity 40, 187–198 (2014). 24485804 10.1016/j.immuni.2013.11.022 PMC4105266

[b34] Gorlova O. *et al.* Identification of novel genetic markers associated with clinical phenotypes of systemic sclerosis through a genome-wide association strategy. PLoS Genet. 7, e1002178 (2011). 21779181 10.1371/journal.pgen.1002178 PMC3136437

[b35] Lessard C. J. *et al.* Identification of IRF8, TMEM39A, and IKZF3-ZPBP2 as susceptibility loci for systemic lupus erythematosus in a large-scale multiracial replication study. Am. J. Hum. Genet. 90, 648–660 (2012). 22464253 10.1016/j.ajhg.2012.02.023 PMC3322228

[b36] Chrabot B. S. *et al.* Genetic variation near IRF8 is associated with serologic and cytokine profiles in systemic lupus erythematosus and multiple sclerosis. Genes Immun. 14, 471–478 (2013). 23965942 10.1038/gene.2013.42 PMC3856198

[b37] Leonard D. *et al.* Coronary heart disease in systemic lupus erythematosus is associated with interferon regulatory factor-8 gene variants. Circ. Cardiovasc. Genet. 6, 255–263 (2013). 23661672 10.1161/CIRCGENETICS.113.000044

[b38] International Multiple Sclerosis Genetics, C. The genetic association of variants in CD6, TNFRSF1A and IRF8 to multiple sclerosis: a multicenter case-control study. PLoS One 6, e18813 (2011). 21552549 10.1371/journal.pone.0018813 PMC3084233

[b39] Nelson N. *et al.* Expression of IFN regulatory factor family proteins in lymphocytes. Induction of Stat-1 and IFN consensus sequence binding protein expression by T cell activation. J. Immunol. 156, 3711–3720 (1996). 8621906

[b40] Zhou Z. Y., Chen S. L., Shen N. & Lu Y. Cytokines and Behcet’s disease. Autoimmun. Rev. 11, 699–704 (2012). 22197901 10.1016/j.autrev.2011.12.005

[b41] Wojdasiewicz P., Poniatowski L. A. & Szukiewicz D. The role of inflammatory and anti-inflammatory cytokines in the pathogenesis of osteoarthritis. Mediators Inflamm. 2014, 561459 (2014). 24876674 10.1155/2014/561459 PMC4021678

[b42] Kim S. H. *et al.* Dual Function of the IRF8 Transcription Factor in Autoimmune Uveitis: Loss of IRF8 in T Cells Exacerbates Uveitis, Whereas Irf8 Deletion in the Retina Confers Protection. J. Immunol. 195, 1480–1488 (2015). 26163590 10.4049/jimmunol.1500653 PMC4530071

[b43] Taniguchi T., Ogasawara K., Takaoka A. & Tanaka N. IRF family of transcription factors as regulators of host defense. Annu. Rev. Immunol. 19, 623–655 (2001). 11244049 10.1146/annurev.immunol.19.1.623

[b44] Fu Q. *et al.* Association of a functional IRF7 variant with systemic lupus erythematosus. Arthritis Rheum. 63, 749–754 (2011). 21360504 10.1002/art.30193 PMC3063317

[b45] Lin L. H., Ling P. & Liu M. F. The potential role of interferon-regulatory factor 7 among Taiwanese patients with systemic lupus erythematosus. J. Rheumatol. 38, 1914–1919 (2011). 21632682 10.3899/jrheum.101004

[b46] Ancient missense mutations in a new member of the RoRet gene family are likely to cause familial Mediterranean fever. The International FMF Consortium. Cell 90, 797–807 (1997). 9288758 10.1016/s0092-8674(00)80539-5

[b47] French F. M. F. C. A candidate gene for familial Mediterranean fever. Nat. Genet. 17, 25–31 (1997). 9288094 10.1038/ng0997-25

[b48] Tasliyurt T., Yigit S., Rustemoglu A., Gul U. & Ates O. Common MEFV gene mutations in Turkish patients with Behcet’s disease. Gene 530, 100–103 (2013). 23973724 10.1016/j.gene.2013.08.026

[b49] Villani A. C. *et al.* Genetic variation in the familial Mediterranean fever gene (MEFV) and risk for Crohn’s disease and ulcerative colitis. PLoS One 4, e7154 (2009). 19784369 10.1371/journal.pone.0007154 PMC2745755

[b50] Arancibia S. A. *et al.* Toll-like receptors are key participants in innate immune responses. Biol. Res. 40, 97–112 (2007). 18064347 10.4067/s0716-97602007000200001

[b51] Ghosh S. & Hayden M. S. New regulators of NF-kappaB in inflammation. Nat. Rev. Immunol. 8, 837–848 (2008). 18927578 10.1038/nri2423

[b52] Yalcin B., Atakan N. & Alli N. The functional role of nuclear factor kappa-kappaB1 -94 ins/del ATTG promotor gene polymorphism in Behcet’s disease: an exploratory study. Clin. Exp. Dermatol. 33, 629–633 (2008). 18616724 10.1111/j.1365-2230.2008.02786.x

[b53] Criteria for diagnosis of Behcet’s disease. International Study Group for Behcet’s Disease. Lancet 335, 1078–1080 (1990). 1970380

[b54] Read R. W. *et al.* Revised diagnostic criteria for Vogt-Koyanagi-Harada disease: report of an international committee on nomenclature. Am. J. Ophthalmol. 131, 647–652 (2001). 11336942 10.1016/s0002-9394(01)00925-4

[b55] Hedrick P. W. Gametic disequilibrium measures: proceed with caution. Genetics 117, 331–341 (1987). 3666445 10.1093/genetics/117.2.331 PMC1203208

