# Supplementary material for: Two Genetic Variations in the IRF8 region are associated with Behçet’s disease in Han Chinese
Source: Sci Rep. 2016 Jan 22;6:19651. doi: 10.1038/srep19651 (PMC4726413; doi:10.1038/srep19651)
Supplement: Supplementary Information [file srep19651-s1.pdf]

Two Genetic Variations in the IRF8 region are associated with Behçet's disease in Han Chinese

Yanni Jiang[1*], Hong Wang[2*], Hongsong Yu[1], Lin Li[1], Dengfeng Xu[1], Shengping Hou[1], Aize Kijlstra[3], Peizeng Yang[1]

[1]The First Affiliated Hospital of Chongqing Medical University, Chongqing Key Laboratory of Ophthalmology and Chongqing Eye Institute, Chongqing, P R China.

[2]Beijing Tongren Eye Center, Beijing Key Laboratory of Ophthalmology and Visual Science, Beijing Tongren Hospital, Capital Medical University, Beijing, P R China.

[3] University Eye Clinic Maastricht, Maastricht, The Netherlands.

*These authors contributed equally to this work

Correspondence to: Professor Peizeng Yang, MD, PhD.

The First Affiliated Hospital of Chongqing Medical University, Chongqing Key Laboratory of Ophthalmology and Chongqing Eye Institute, Chongqing, P. R. China, 400016;

Phone: +8623 89012851;

FAX: +8623 89012851;

Email: peizengycmu@126.com

Supplementary Figure 1

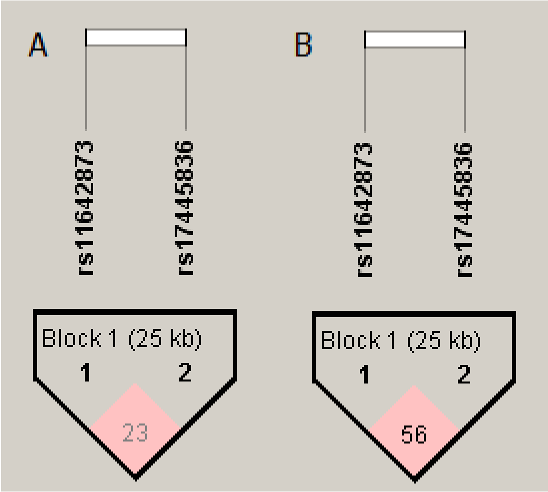

Supplementary Figure 2

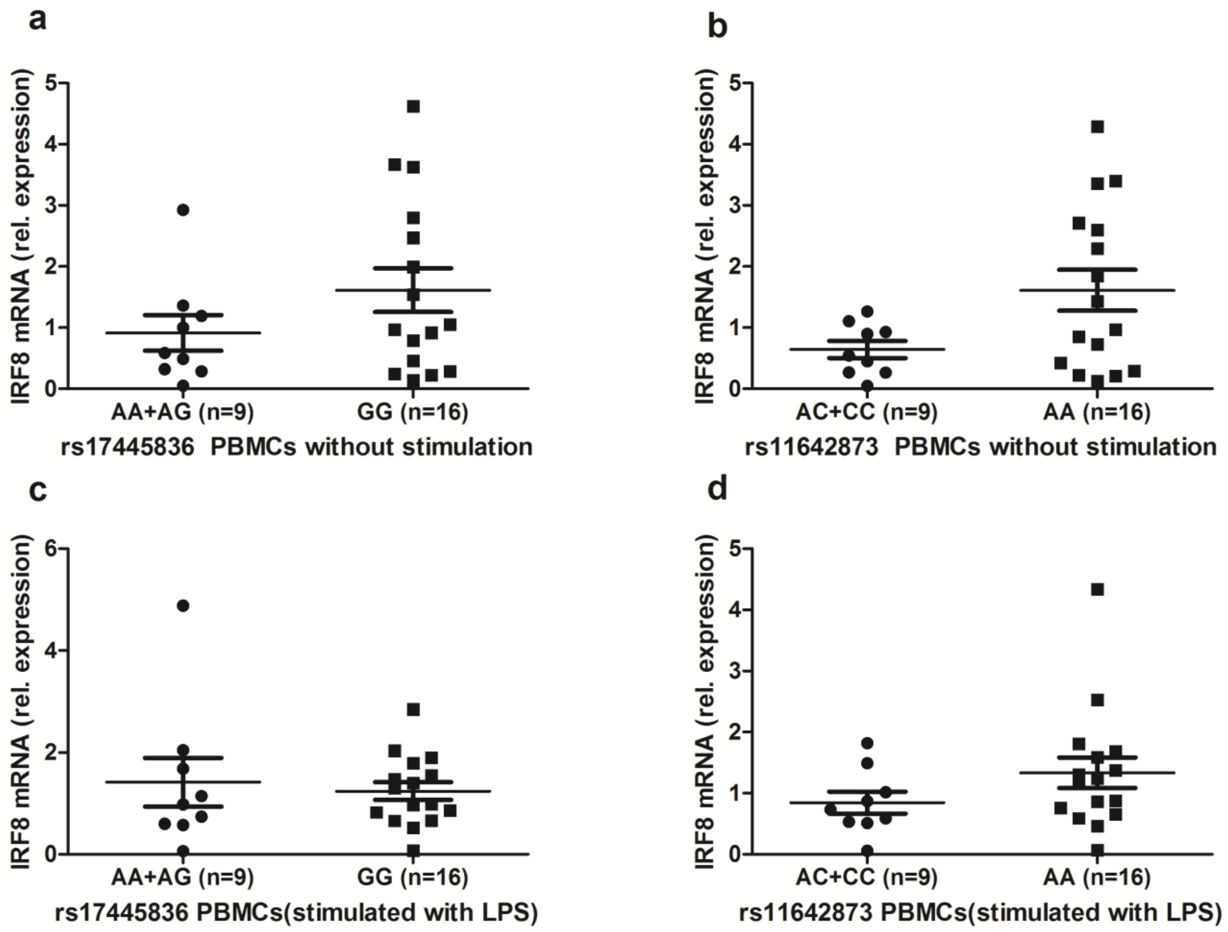

Supplementary Figure Legends

Supplementary Figure 1. Linkage disequilibrium (LD) of rs11642873 and rs17445836 performed by Haploview4.2 software in BD patients and normal controls enrolled in present study. (A): LD measure of r^2=0.23; and (B): LD

measure of D'=0.56.

Supplementary Figure 2. The influence of rs17445836 and rs11642873 on the relative expression of IRF8. The expression of IRF8 in unstimulated PBMCs obtained from genotyped healthy individuals with diverse genotypes of rs17445836 (a) and rs11642873 (b). The expression of IRF8 in PBMCs following LPS stimulation comparing the various genotypes of rs17445836 (c) and rs11642873 (d). The mRNA expression of IRF8 was not significantly different between genotypes (Pc>0.05). Data are shown as mean ±SD. Pc: Bonferroni corrected p value, multiplied by 3.

Supplementary Table 1.Primers and restriction enzymes utilized for RFLP analysis of IRF3, IRF7, IRF8, MYD88, P50 and TRIM20 gene polymorphisms.

Gene	SNPs ID	Primers	Restriction enzymes	Length of the target segments (bp)
IRF3	rs2304204	5'CAATTTGCATGTGACGCTCCC3'<br>5'TCTTCCGCGTTACCTACGATG3'	PvuI*	294bp(146+148)
IRF7	rs1061501	5'GTCGCTTCGTGATGCCGCG3'<br>5'ATGTTCCCCTTTGCCCAAGC3'	SacII*	147bp(18+129)
IRF8	rs11644034	5'AGCCACCTTTCCTATCATGCC3'<br>5'GGTCCCATGCACCTTAACCAC3'	EcoO109I*	344bp(89+255)
	rs925994	5'CCAAGCTACTCTTAAGGATGCCATTATT3'<br>5'GACTAAACCTCGTGCAGCCT3'	HpyF10VI*	102bp(72+26)
	rs11648084	5'GGCCATTTGCAGAACATTCCGTTGA3'<br>5'TGCACCTTAACCACAGTTGGCA3'	HincII*	121bp(23+99)
	rs2280381	5'TTGAAGGAACTAAAATTTTGTCTGGGCTAC3'<br>5' CCAAGCATCTGTACCTGGCA3'	TaiI*	228bp(32+196)
	rs11642873	5'CCGTTTCAGGTTCACAGCAAGA3'<br>5'CTGGGTGATAATGAAGTGTCCGTG3'	BtgI**	172bp(24+148)
	rs17445836	5'CCAGGTGGAAATGGAGGT3'<br>5' CAGGCACAGGGCTAAGAA3'	PstI*	282bp(204+78)
MYD88	rs7744	5'AATTGGGCACTCATCTATTCC3'<br>5'GTATAAATTGCTCTGGGAAGG3'	BfaI*	193(90+103)
NF-κB1	rs3774959	5'CTCCTTAACTGCTCCAGCCC3'<br>5' TTGTTTTGTTTTCATCTTTCTGTGTCT3'	BstMAI*	183(155+33)
TRIM20	rs224204	5' GACCTGGGAGGGAGATGATCCCAA3'<br>5' GCACGGGACACAATGCACTC3'	Bse8I*	227bp(206+21)
	rs224217	5'ACCATCTTTAGAATCCAAGGGGTTTATGT3'<br>5' TGGGACCGCCTGTGTATCAT3'	RsaI*	300bp(271+29)
	rs224225	5' AGAATATTCCACACAAGAAAACGGCACATA3'<br>5'TGCGTTTGCTCAGGGGCTT3'	NdeI*	209bp(28+181)

* Fermentas China Co. Ltd., Shenzhen, China, **New England Biolabs China Co. Ltd., Beijing, China.

Supplementary Table 2. Clinical features, age and gender distribution in investigated patients with BD and patients with VKH

Extraocular findings	Total	%
Patients with BD	1081	
Mean age ±SD	33.8 ±9.1	
Male	847	78.4
Female	233	21.6
Uveitis	1081	100
Oral ulcer	1081	100
Skin lesions	844	78.1
Genital ulcer	620	57.4
Hypopyon	284	26.3
Arthritis	206	19.1
Positive pathergy test	200	18.5
Patients with VKH	384	
Mean age ±SD	33.5 ±10.6	
Male	201	52.3
Female	183	47.7
Uveitis	384	100
Tinnitus	165	43.0
Alopecia	153	39.8
Headache	144	37.5
Gray hair	137	35.7
Vitiligo	70	28.3
Scalp allergy	61	15.9
Nuchal rigidity	37	9.6
Controls	2066	
Mean age ±SD	33.8 ±8.1	
Male	1077	52.1
Female	989	47.9

BD: Behçet's disease; VKH: Vogt–Koyanagi–Harada.

Supplementary Table 3. Frequencies of genotypes and alleles of IRF3, IRF7, IRF8, MYD88, P50 and TRIM20 polymorphisms in patients with BD and controls.

**Gene**	**SNPs**	**Genotype**	**Case**	**Control**	**P value**	**Pc value**	**OR(95%CI)**
		Allele	N(freq.)	N(freq.)			
**IRF3**	rs2304204	A A	216(0.562)	307(0.533)	0.368	NS	1.127(0.869-1.461)
		A G	149(0.388)	230(0.399)	0.726	NS	0.954(0.732-1.243)
		G G	19(0.049)	39(0.068)	0.246	NS	0.717(0.408-1.260)
		G	187(0.243)	308(0.267)	0.241	NS	0.882(0.715-1.088)
		A	581(0.757)	844(0.733)	0.241	NS	1.134(0.919-1.399)
**IRF7**	rs1061501	A A	199(0.518)	254(0.444)	0.024	NS	1.347(1.039-1.745)
		A G	151(0.393)	268(0.469)	0.021	NS	0.735(0.565-0.956)
		G G	34(0.089)	50(0.087)	0.952	NS	1.014(0.643-1.600)
		G	219(0.285)	368(0.322)	0.09	NS	0.841(0.689-1.027)
		A	549(0.715)	776(0.678)	0.09	NS	1.189(0.973-1.452)
**IRF8**	rs11644034	AA	6(0.016)	4(0.007)	0.194	NS	2.270(0.636-8.097)
		AG	76(0.198)	127(0.220)	0.401	NS	0.872(0.634-1.200)
		GG	302(0.786)	445(0.773)	0.612	NS	1.084(0.793-1.482)
		G	680(0.885)	1017(0.883)	0.861	NS	1.026(0.771-1.365)
		A	88(0.115)	135(0.117)	0.861	NS	0.975(0.733-1.297)
	rs925994	T T	21(0.055)	14(0.024)	0.014	NS	2.322(1.166-4.625)
		G T	116(0.302)	183(0.318)	0.609	NS	0.930(0.703-1.229)
		G G	247(0.643)	379(0.658)	0.638	NS	0.937(0.715-1.228)
		G	610(0.794)	941(0.817)	0.219	NS	0.866(0.688-1.090)
		T	158(0.206)	211(0.183)	0.219	NS	1.155(0.918-1.454)
	rs11648084	C C	131(0.346)	148(0.258)	0.004	NS	1.520(1.146-2.017)
		C T	163(0.430)	304(0.530)	0.003	NS	0.670(0.516-0.870)
		T T	85(0.224)	122(0.213)	0.667	NS	1.071(0.783-1.465)
		C	425(0.561)	600(0.523)	0.103	NS	1.166(0.969-1.402)
		T	333(0.439)	548(0.477)	0.103	NS	0.858(0.713-1.032)
	rs2280381	A A	301(0.784)	424(0.736)	0.092	NS	1.300(0.598-1.765)
		A G	78(0.203)	144(0.250)	0.092	NS	0.765(0.560-1.045)
		G G	5(0.013)	8(0.014)	0.909	NS	0.937(0.304-2.885)
		G	88(0.115)	160(0.139)	0.12	NS	0.802(0.608-1.059)
		A	680(0.885)	992(0.861)	0.12	NS	1.246(0.944-1.645)
**MYD88**	rs7744	A A	170(0.445)	229(0.399)	0.157	NS	1.208(0.930-1.570)
		A G	169(0.442)	281(0.490)	0.153	NS	0.827(0.638-1.073)
		G G	43(0.113)	64(0.111)	0.959	NS	1.011(0.671-1.523)
		G	255(0.334)	409(0.356)	0.311	NS	0.905(0.746-1.098)
		A	509(0.666)	739(0.644)	0.311	NS	1.105(0.911-1.340)
**NF-κB1**	rs3774959	A A	72(0.188)	106(0.184)	0.892	NS	1.023(0.734-1.426)
		A G	197(0.513)	294(0.510)	0.937	NS	1.010(0.780-1.308)
		G G	115(0.299)	176(0.306)	0.841	NS	0.972(0.733-1.287)
		G	427(0.556)	646(0.561)	0.836	NS	0.981(0.816-1.179)
		A	341(0.444)	506(0.439)	0.836	NS	1.020(0.848-1.225)

**TRIM20**	rs224204	C C	58(0.151)	101(0.175)	0.321	NS	0.837(0.588-1.190)
		C T	189(0.492)	305(0.530)	0.257	NS	0.861(0.665-1.115)
		T T	137(0.357)	170(0.295)	0.045	NS	1.325(1.006-1.744)
		C	305(0.397)	507(0.440)	0.062	NS	0.838(0.696-1.009)
		T	463(0.603)	645(0.560)	0.062	NS	1.193(0.991-1.437)
	rs224217	CC	244(0.635)	404(0.701)	0.033	NS	0.742(0.564-0.976)
		CT	134(0.349)	158(0.274)	0.014	NS	1.418(1.073-1.873)
		TT	6(0.016)	14(0.024)	0.356	NS	0.637(0.243-1.673)
		C	622(0.810)	966(0.839)	0.104	NS	0.820(0.646-1.042)
		T	146(0.190)	186(0.161)	0.104	NS	1.219(0.960-1.548)
	rs224225	C C	4(0.010)	9(0.016)	0.494	NS	0.663(0.203-2.169)
		C T	130(0.339)	168(0.292)	0.124	NS	1.243(0.942-1.640)
		T T	250(0.651)	399(0.693)	0.177	NS	0.828(0.629-1.089)
		C	138(0.180)	186(0.161)	0.296	NS	1.138(0.893-1.449)
		T	630(0.820)	966(0.839)	0.296	NS	0.879(0.690-1.120)

Pc: Bonferroni corrected p value, multiplied by 39 /genotype and 13 /allele. NS: not significant.

Supplementary Table 4. Frequencies of genotypes and alleles of IRF3, IRF7, IRF8, MYD88, P50 and TRIM20 polymorphisms in patients with VKH and controls.

Gene	SNPs	Genotype<br>Allele	Case<br>N(freq.)	Control<br>N(freq.)	P value	Pc value	OR(95%CI)
**IRF3**	rs2304204	A A	177(0.461)	307(0.533)	0.029	NS	0.749(0.578-0.971)
		A G	171(0.445)	230(0.399)	0.157	NS	1.208(0.930-1.568)
		G G	36(0.094)	39(0.068)	0.141	NS	1.424(0.888-2.285)
		G	243(0.316)	308(0.267)	0.02	NS	1.268(1.038-1.550)
		A	525(0.684)	844(0.733)	0.02	NS	0.788(0.645-0.963)
**IRF7**	rs1061501	A A	185(0.482)	254(0.444)	0.251	NS	1.164(0.898-1.509)
		A G	166(0.432)	268(0.469)	0.27	NS	0.864(0.666-1.121)
		G G	33(0.086)	50(0.087)	0.937	NS	0.982(0.620-1.555)
		G	232(0.302)	368(0.322)	0.365	NS	0.913(0.749-1.112)
		A	536(0.698)	776(0.678)	0.365	NS	1.096(0.899-1.335)
**IRF8**	rs11644034	AA	4(0.010)	4(0.007)	0.562	NS	1.505(0.374-6.055)
		AG	76(0.198)	127(0.220)	0.401	NS	0.872(0.634-1.200)
		GG	304(0.792)	445(0.773)	0.484	NS	1.119(0.817-1.531)
		G	684(0.891)	1017(0.883)	0.598	NS	1.081(0.810-1.443)
		A	84(0.109)	135(0.117)	0.598	NS	0.925(0.693-1.235)
	rs925994	T T	9(0.023)	14(0.024)	0.931	NS	0.963(0.413-2.249)
		G T	98(0.255)	183(0.318)	0.037	NS	0.736(0.551-0.982)
		G G	277(0.721)	379(0.658)	0.039	NS	1.346(1.015-1.784)
		G	652(0.849)	941(0.817)	0.067	NS	1.260(0.984-1.614)
		T	116(0.151)	211(0.183)	0.067	NS	0.793(0.619-1.016)
	rs11648084	C C	128(0.337)	148(0.258)	0.008	NS	1.462(1.101-1.941)
		C T	162(0.426)	304(0.530)	0.002	NS	0.660(0.508-0.857)
		T T	90(0.237)	122(0.213)	0.377	NS	1.150(0.844-1.567)
		C	418(0.550)	600(0.523)	0.241	NS	1.116(0.929-1.342)
		T	342(0.450)	548(0.477)	0.241	NS	0.896(0.745-1.077)
	rs2280381	A A	287(0.747)	424(0.736)	0.696	NS	1.061(0.789-1.425)
		A G	94(0.245)	144(0.250)	0.855	NS	0.972(0.721-1.312)
		G G	3(0.008)	8(0.014)	0.386	NS	0.559(0.147-2.121)
		G	100(0.130)	160(0.139)	0.586	NS	0.928(0.710-1.214)
		A	668(0.870)	992(0.861)	0.586	NS	1.077(0.824-1.409)
	rs11642873	A A	302(0.805)	433(0.758)	0.089	NS	1.318(0.958-1.815)
		A C	73(0.195)	133(0.233)	0.163	NS	0.796(0.578-1.097)
		C C	0	5(0.009)	0.069	NS	1.009(1.001-1.017)
		A	677(0.903)	999(0.875)	0.062	NS	1.328(0.985-1.789)
		C	73(0.097)	143(0.125)	0.062	NS	0.753(0.559-1.015)
	rs17445836	A A	0	1(0.002)	0.414	NS	1.002(0.998-1.005)
		A G	68(0.178)	85(0.151)	0.271	NS	1.217(0.858-1.725)
		G G	315(0.822)	478(0.848)	0.305	NS	0.833(0.588-1.181)
		G	698(0.911)	1041(0.923)	0.364	NS	0.858(0.616-1.195)
		A	68(0.089)	87(0.077)	0.364	NS	1.166(0.837-1.624)

**MYD88**	rs7744	A A	146(0.382)	229(0.399)	0.603	NS	0.932(0.715-1.215)
		A G	190(0.497)	281(0.490)	0.812	NS	1.032(0.979-1.337)
		G G	46(0.120)	64(0.111)	0.672	NS	1.091(0.729-1.633)
		G	282(0.369)	409(0.356)	0.567	NS	1.057(0.874--1.279)
		A	482(0.631)	739(0.644)	0.567	NS	0.946(0.782-1.144)
**NF-κB1**	rs3774959	A A	62(0.161)	106(0.184)	0.367	NS	0.854(0.605-1.204)
		A G	189(0.492)	294(0.510)	0.58	NS	0.930(0.718-1.204)
		G G	133(0.346)	176(0.306)	0.185	NS	1.204(0.915-1.585)
		G	455(0.592)	646(0.561)	0.169	NS	1.139(0.946-1.370)
		A	313(0.408)	506(0.439)	0.169	NS	0.878(0.730-1.057)
**TRIM20**	rs224204	C C	70(0.182)	101(0.175)	0.783	NS	1.048(0.749-1.468)
		C T	196(0.510)	305(0.530)	0.562	NS	0.926(0.715-1.200)
		T T	118(0.307)	170(0.295)	0.687	NS	1.059(0.800-1.403)
		C	336(0.438)	507(0.440)	0.91	NS	0.989(0.823-1.189)
		T	432(0.562)	645(0.560)	0.91	NS	1.011(0.841-1.215)
	rs224217	CC	258(0.672)	404(0.701)	0.333	NS	0.872(0.660-1.151)
		CT	117(0.305)	158(0.274)	0.308	NS	1.159(0.873-1.540)
		TT	9(0.023)	14(0.024)	0.931	NS	0.963(0.413-2.249)
		C	633(0.824)	966(0.839)	0.41	NS	0.903(0.708-1.151)
		T	135(0.176)	186(0.161)	0.41	NS	1.108(0.868-1.413)
	rs224225	C C	2(0.005)	9(0.016)	0.137	NS	0.330(0.071-1.535)
		C T	127(0.331)	168(0.292)	0.199	NS	1.200(0.909-1.585)
		T T	255(0.664)	399(0.693)	0.351	NS	0.877(0.665-1.156)
		C	131(0.171)	186(0.161)	0.598	NS	1.068(0.836-1.364)
		T	637(0.829)	966(0.839)	0.598	NS	0.936(0.733-1.196)

Pc: Bonferroni corrected p value, multiplied by 39 /genotype and 13 /allele. NS: not significant.

Supplementary Table 5. Frequencies of genotypes and alleles of rs1800871/IL-10 and rs3021094/IL-10 polymorphisms of 25 unrelated genotyped healthy individuals in rs11642873.

SNPs	Genotype	AC+CC cases N(freq.)	AA cases N(freq.)	P value	Pc value	OR(95%CI)
rs1800871	C C	2(0.222)	2(0.125)	0.602	NS	2.000(2.231-17.338)
	C T	3(0.333)	4(0.250)	0.673	NS	1.500(0.251-8.977)
	T T	4(0.444)	10(0.625)	0.434	NS	0.480(0.091-2.523)
	C	7(0.389)	8(0.250)	0.348	NS	1.909(0.552-6.599)
	T	11(0.611)	24(0.750)	0.348	NS	0.524(0.152-1.811)
rs3021094	A A	2(0.222)	10(0.625)	0.097	NS	0.171(0.026-1.111)
	A C	4(0.444)	3(0.188)	0.205	NS	3.467(0.563-21.350)
	C C	3(0.333)	3(0.188)	0.630	NS	2.167(0.334-14.057)
	A	8(0.444)	23(0.719)	0.073	NS	0.313(0.094-1.047)
	C	10(0.556)	9(0.281)	0.073	NS	3.194(0.955-10.686)

Pc: Bonferroni corrected p value, multiplied by 6 /genotype and 2 /allele. NS: not significant.

Supplementary Table 6. Frequencies of genotypes and alleles of rs1800871/IL-10 and rs3021094/IL-10 polymorphisms of 25 unrelated genotyped healthy individuals in rs17445836.

SNPs	Genotype	AA+AG cases N(freq.)	GG cases N(freq.)	P value	Pc value	OR(95%CI)
rs1800871	C C	3(0.333)	3(0.188)	0.630	NS	2.167(0.334-14.057)
	C T	4(0.444)	5(0.313)	0.671	NS	1.760(0.326-9.510)
	T T	2(0.222)	8(0.500)	0.229	NS	0.286(0.045-1.821)
	C	10(0.556)	11(0.344)	0.232	NS	2.386(0.732-7.780)
	T	8(0.444)	21(0.656)	0.232	NS	0.419(0.129-1.366)
rs3021094	A A	3(0.333)	9(0.563)	0.411	NS	0.389(0.071-2.133)
	A C	3(0.333)	3(0.188)	0.630	NS	2.167(0.334-14.057)
	C C	3(0.333)	4(0.250)	0.673	NS	1.500(0.251-8.977)
	A	9(0.500)	21(0.656)	0.370	NS	0.524(0.161-1.700)
	C	9(0.500)	11(0.344)	0.370	NS	1.909(0.588-6.196)

Pc: Bonferroni corrected p value, multiplied by 6 /genotype and 2 /allele. NS: not significant.
